# UV reflective properties of magnesium oxide increase attraction and probing behavior of Asian citrus psyllids (Hemiptera: Liviidae)

**DOI:** 10.1038/s41598-020-58593-4

**Published:** 2020-02-05

**Authors:** Justin George, Thomson M. Paris, Sandra A. Allan, Stephen L. Lapointe, Lukasz L. Stelinski

**Affiliations:** 1 United States Department of Agriculture, Agricultural Research Service, 2001 South Rock Road, Fort Pierce, FL 34945 USA; 2 University of Florida, Entomology and Nematology Department, Citrus Research and Education Center, 700 Experiment Station Rd., Lake Alfred, FL 33850 USA; 3 United States Department of Agriculture, Agricultural Research Service, 1700 SW 23rd drive, Gainesville, FL 32608 USA

**Keywords:** Zoology, Entomology, Behavioural ecology

## Abstract

Asian citrus psyllid (*Diaphorina citri)* vectors the bacterium *Candidatus* Liberibacter asiaticus, the causal pathogen of citrus greening disease that is devastating citrus industries worldwide. Suppressing psyllid populations is crucial to prevent disease spread. An attract-and-kill trap based on psyllid behavior would fill a niche for monitoring and control. To optimize visual attraction of psyllids, the ultraviolet (UV) reflective properties of magnesium oxide (MgO) and/or barium sulfate (BaSO_4_) were assessed for potential application to a trap surface. Under low UV, high UV and natural sunlight conditions, the reflectance, attraction, and probing behaviors of psyllids were evaluated on surfaces containing magnesium oxide or barium sulfate. Magnesium oxide added to yellow sticky traps enhanced visual response of *D. citri*. Probing assays demonstrated that magnesium oxide alone or as a mixture with a phagostimulant blend, increased the UV reflectance of substrates, as well as, attraction and probing by psyllids. Results demonstrated that psyllids respond to both short (UV) and long (yellow) wavelengths during orientation, and that these inert compounds can increase UV reflectance and improve attractiveness of an attract-and-kill device.

## Introduction

Visual and olfactory cues play a crucial role in insect orientation and successful host selection. Understanding the visual, olfactory, tactile, and gustatory cues that insects use to select their hosts is essential to the development of sustainable control strategies against insects, particularly plant disease vectors. Asian citrus psyllids (*Diaphorina citri*) are vectors of the putative bacterial pathogen, *Candidatus* Liberibacter asiaticus (CLas) that causes huanglongbing (HLB) aka citrus greening disease. Multiple sensory cues are used by *D. citri* for host plant finding including visual, olfactory and tactile cues^1–6^. Many insects discriminate between wavelengths of light reflecting from visual targets independent of their intensity to make decisions when selecting hosts^7^. Both positive phototactic and negative geotactic behaviors have been demonstrated for *D. citri*^2,8–10^. Attraction of phytophagous hemipterans to yellow and green targets with a particularly strong preference for yellow visual targets has been documented for aphids^11,12^, whiteflies^13–15^ and psyllids^16–18^. In contrast, psyllids, as well as aphids, exhibit only weak chemotactic responses in the absence of visual cues^1,2,19^.

Behavioral responses of *D. citri* adults to visual cues has been primarily investigated using yellow and lime green^17,18,20^ colored traps in field studies and to yellow, green and ultraviolet (UV) light-emitting diodes (LEDs) in laboratory assays^21^. Filters transmitting a broad spectrum of wavelengths revealed that *D. citri* adults respond more to both long (green, yellow) and short (UV) wavelengths of light than to long wavelengths alone^8^. Generally, Hemiptera are not attracted to visual targets that reflect or emit light <400 nm or >600 nm^22,23^. However, attraction to longer wavelength red light occurs in eucalyptus psyllids, *Anoeconeossa bundoorensis* and *Glycaspis brimblecombei*, which specialize on red anthocyanic leaves^24^. Furthermore, attraction of *D. citri* adults to transparent visual targets comprised of green or yellow filters can be enhanced with addition of UV light (<400 nm)^8^.

There is significant variation in the number of spectral receptor classes and photopigments among insects. The green peach aphid, *Myzus persicae*, for example, exhibits maximum sensitivities at 320–330, 440–480 and 530 nm^25^, as measured electrophysiologically. Similarly, the cabbage aphid, *Brevicoryne brassicae*, displays two peaks of spectral sensitivity at 350 and 520–530 nm^26^. Extracellular measurements from *D*. *citri* eyes indicated peaks of sensitivity in the UV, blue, and yellow/green regions^27^. Many invertebrate photopigments exhibit a minor UV peak of absorption as part of photo-transduction. However, certain species possess spectral receptors that exhibit peak sensitivity in the UV spectrum^28,29^. Examples include the butterflies, which rely on short and long wavelengths to mediate interspecific and intraspecific sexual selection behaviors^30^. In some cases, it has been realized that reflection of UV light from substrates can have practical applications for pest control by disrupting normal insect behaviors associated with host plant finding. A prominant example for management of day flying insects is use of metalized mulches as a form of insect repellent. Such mulches can be deployed underneath citrus trees to protect them from *D. citri* infestation by taking advantage of the UV component of visual orientation and effectively repelling these insects away from their host plants^31^.

Yellow-colored traps coated in adhesive are widely used for monitoring psyllids, but these traps become ineffective after brief periods of deployment as they become fouled and require frequent replacement. Efforts to improve sensitivity of yellow sticky traps^32^ using baits such as olfactory lures based on plant and/or *D. citri* volatiles have not been promising^2,33^. Visual cues may play a dominant role in host location by *D. citri* adults and therefore may be more important than olfactory cues in the design of effective traps or attract-and-kill devices^2,32,33^. More detailed insight into visually driven behaviors of *D. citri* may facilitate improvement of surveillance and management for this species.

Asian citrus psyllids are strongly attracted to visual targets that emit combined UV and yellow or green wavelengths^8^. When using UV filters in combination with different colors, removal of the UV light component reduces attraction to each color^8^. The main objective of this research was to investigate the UV reflective properties of magnesium oxide and barium sulfate, and their practical use as additives to increase attraction of *D. citri* adults to an attract-and-kill device and subsequent probing on a wax substrate. Attraction to and probing behaviors of *D. citri* on SPLAT (Specialized Pheromone and Lure Application Technology) containing magnesium oxide and barium sulfate were quantified under low UV, high UV and natural sunlight conditions. We hypothesized that magnesium oxide and barium sulfate would increase attraction of *D. citri* adults to an attract-and-kill device because of increased UV reflectance. The impact of adding varying levels of magnesium oxide on visual attraction of *D. citri* to yellow sticky traps was also examined. The practical application of using magnesium oxide and barium sulfate as UV reflectants along with other components, as part of an attract-and-kill device for Asian citrus psyllid management are discussed.

## Results

### Measurement of UV reflectance from surfaces of magnesium oxide and barium sulfate

The irradiance spectral measurements for the solar and artificial light sources showed the difference in UV light emissions and how they might influence the attraction and behavior of *D. citri* (Fig. S1). The spectrometer measurements of the three artificial light sources used in the SPLAT bioassays demonstrated that the metal halide lamp provided the most ultraviolet radiation followed by the standard fluorescent lamp (Figs. S1, S2). Unsurprisingly, sunlight radiated more ultraviolet light and exhibited higher intensity than any other light source measured (Figs. S1, S2).

**Figure 1:**
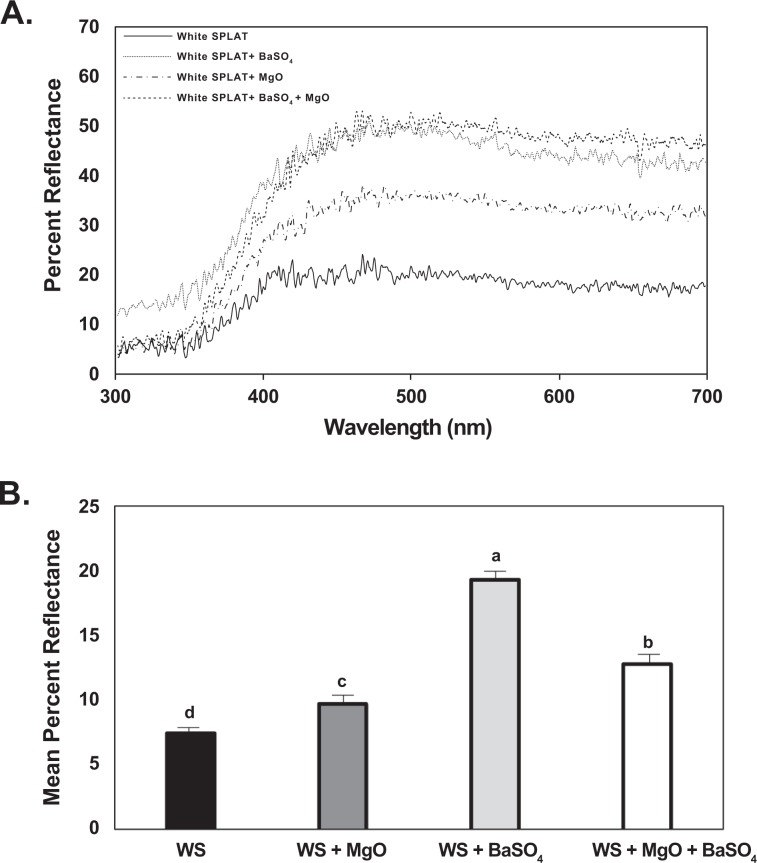
(**A**) Reflectance spectra of white SPLAT beads treatments used in the bioassays. Reflectance spectra of the following visual targets are depicted using a Deuterium-Tungsten Halogen light source. 1. White SPLAT (solid line), 2. White SPLAT + MgO (dotted line), 3. White SPLAT + BaSO_4_ (dash and dot line), 4. White SPLAT + MgO + BaSO_4_ (dashed line). (**B**) Mean percent reflectance spectra of white sticky traps used in the bioassays with respect to ultraviolet radiation measured using a Deuterium-Tungsten Halogen light source. The mean ultraviolet irradiance percent reflectance spectra is depicted between a range of 300 to <400 nm. Means were compared by Tukey’s HSD following a significant ANOVA. Treatments having no letters in common are significantly different (*α* = 0.05).

**Figure 2:**
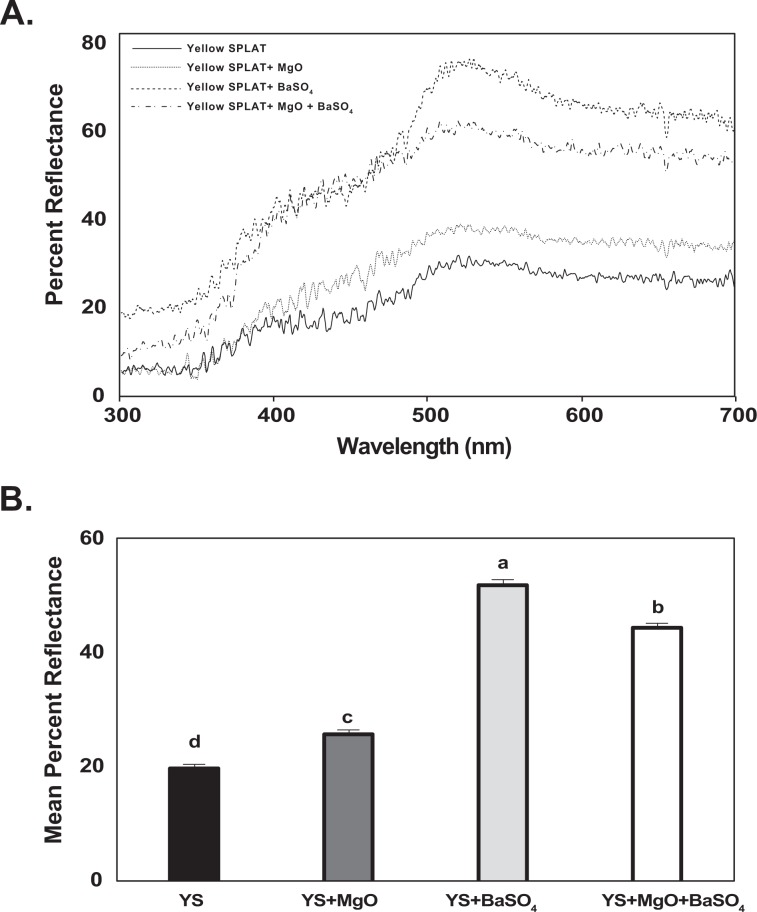
(**A**) Reflectance spectra of yellow SPLAT beads treatments used in the bioassays. Reflectance spectra of the following visual targets are depicted using a Deuterium-Tungsten Halogen light source. 1. Yellow SPLAT (solid line), 2. Yellow SPLAT + MgO (dotted line), 3. Yellow SPLAT + BaSO_4_ (dashed line), 4. Yellow SPLAT + MgO + BaSO_4_ (dash and dot line). (**B**) Mean percent reflectance spectra of yellow SPLAT beads used in the bioassays with respect to ultraviolet radiation measured using a Deuterium-Tungsten Halogen light source. The mean ultraviolet irradiance percent reflectance spectra are depicted between a range of 300 to <400 nm. Means were compared by Tukey’s HSD following a significant ANOVA. Treatments having no letters in common are significantly different (*α* = 0.05).

The reflectance spectra of different SPLAT treatments containing magnesium oxide and barium sulfate were measured. Addition of magnesium oxide and barium sulfate to white (Fig. 1A,B) and yellow SPLAT (Fig. 2A,B) visual targets increased percent reflectance from the surface of SPLAT beads. Barium sulfate had a greater impact on increasing reflectance of both yellow and white targets than magnesium oxide (Figs. 1A, 2A). The addition of barium sulfate, and to a lesser degree magnesium oxide, resulted in increased percent ultraviolet reflectance of the visual targets (Figs. 1B, 2B). The effect of either magnesium oxide or barium sulfate on variation in percent ultraviolet reflectance could be modified by adjusting the quantity of either added to SPLAT. The powdered forms of both compounds had a similar effect on percent reflectance (Fig. S3). Spectrometer measurements of magnesium oxide and barium sulfate showed that they have a reflectance of 93% and 77% in the 350–400 nm UV range, respectively. The highly reflective properties of these compounds suggested either could increase attraction of *D. citri* towards SPLAT containing these compounds.

### Visual attraction of adult psyllids to yellow sticky cards with different levels of magnesium oxide

**Figure 3:**
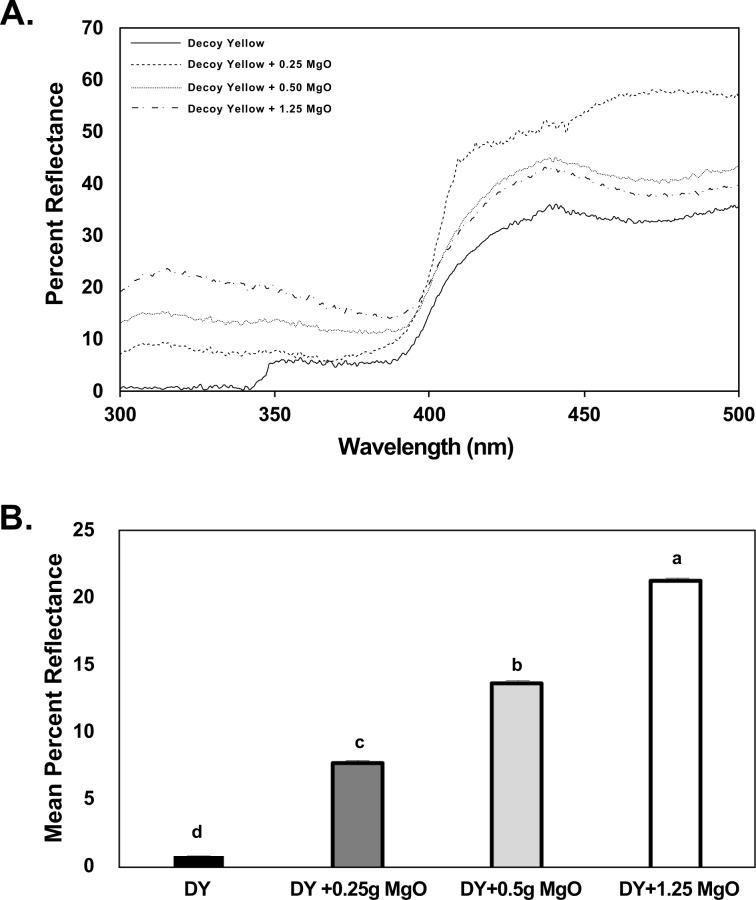
(**A**) Reflectance spectra of decoy yellow sticky traps (DY) used in the bioassays. Reflectance spectra of the following visual targets are depicted using a Deuterium-Tungsten Halogen light source. 1. Yellow SPLAT (solid line), 2. Yellow SPLAT + MgO (dotted line) 3. Yellow SPLAT + BaSO_4_ (dashed line), 4. Yellow SPLAT + MgO + BaSO_4_ (dash and dot line). (**B**) Mean percent reflectance spectra of white sticky traps used in the bioassays with respect to ultraviolet radiation measured using a Deuterium-Tungsten Halogen light source. The mean ultraviolet irradiance percent reflectance spectra are depicted between a range of 300 to <400 nm. Means were compared by Tukey’s HSD following a significant ANOVA. Treatments having no letters in common are significantly different (*α* = 0.05).

Overall, addition of different amounts of magnesium oxide increased the reflectance spectra from yellow sticky card surfaces (Fig. 3A). As was the case with the SPLAT beads, addition of magnesium oxide altered the reflectance of yellow sticky traps most noticeably in the 300-400 nm UV range (Fig. 3B). Percent reflectance of yellow sticky trap surfaces increased with increasing amount of magnesium oxide added (Fig. 3A,B).

**Figure 4:**
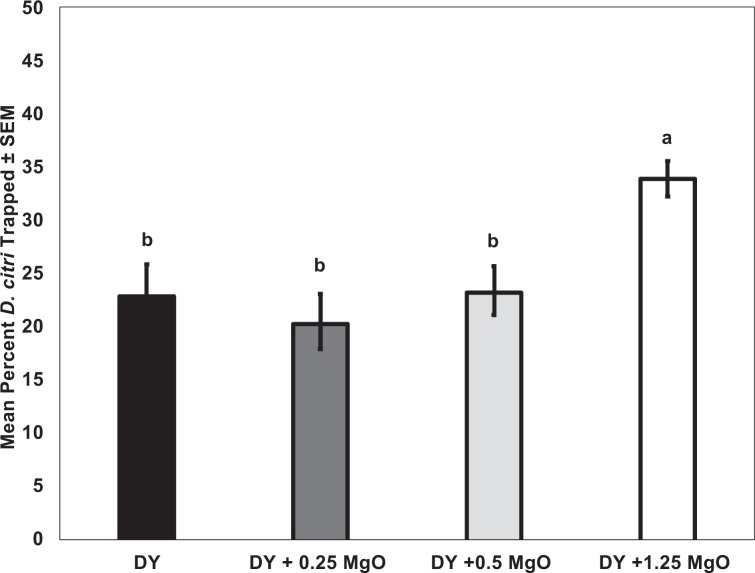
Mean percentage of adult *Diaphorina citri* captured on decoy yellow sticky traps (DY) in a laboratory bioassay. Each bioassay contained four types of sticky traps Decoy yellow (DY), DY + 0.25 g of MgO, DY + 0.5 g MgO, DY + 1.25 g MgO. Means were compared by Tukey’s HSD following a significant ANOVA. Treatments having no letters in common are significantly different (*α* = 0.05).

To study the effect of magnesium oxide on trap catch of yellow sticky traps, behavioral choice assays were performed. More adult *D. citri* were collected on yellow sticky traps with the highest quantity of magnesium oxide (1.25 g) (*F*_*3,35*_ = 14.5; *P* < 0.001, *n* = 12) (Fig. 4). This preference of *D. citri* for the yellow sticky trap with highest reflectance indicated that an achromatic cue is an important component that elicits attraction (Fig. 3A,B).

### Effect of magnesium oxide and barium sulfate on probing by *D. citri* under different UV conditions

**Figure 5:**
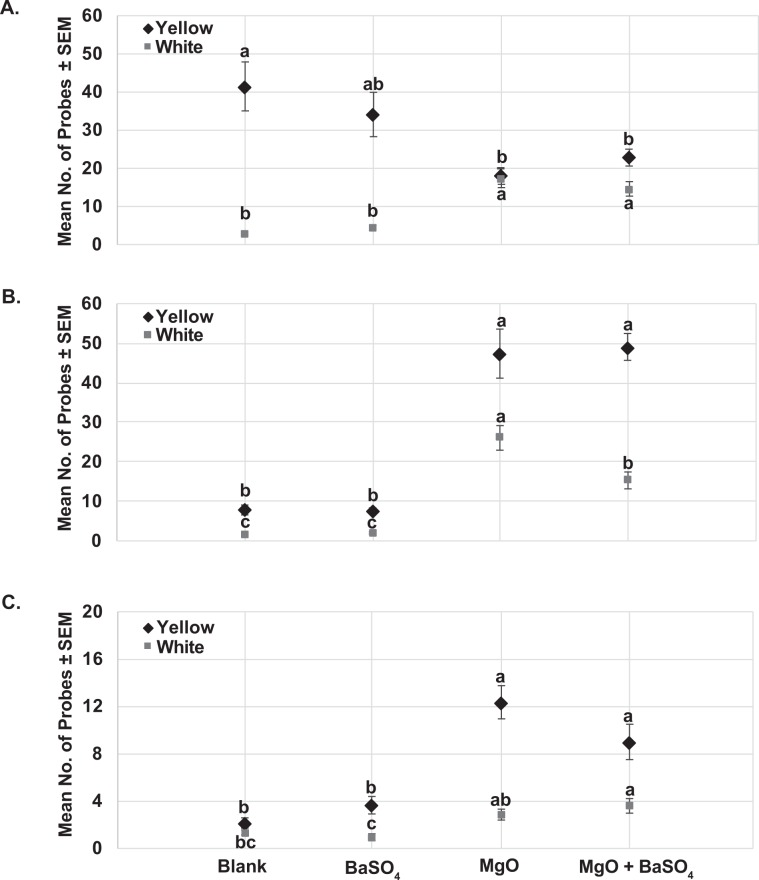
Probing behavior of psyllid adults on yellow and white SPLAT beads containing magnesium oxide (MgO) or barium sulfate (BaSO_4_) or a combination of MgO + BaSO_4_ under different UV light conditions. (**A**) Mean number of probes (salivary sheaths) ± SEM produced by psyllids on white and yellow SPLAT beads under low UV (fluorescent) light conditions (**B**) Mean number of probes ± SEM produced by psyllids on white and yellow SPLAT beads under UV (UV chamber) conditions. (**C**) Mean number of probes ± SEM produced by psyllids on white and yellow SPLAT beads under unfiltered natural sunlight conditions. Means analyzed by one-way ANOVA followed by Tukey’s HSD. Treatments having no letters in common are significantly different (*α* = 0.05) within the white SPLAT treatment and yellow SPLAT treatments.

More salivary sheaths were counted on yellow SPLAT compared with white SPLAT in all experiments (*F*_7,159_ = 62.7; *P* = < 0.0001, *n* = 20) (Table 1). Under standard fluorescent light conditions (low UV) (Fig. 5A), significantly more salivary sheaths were present on blank yellow (42 ± 6 sheaths/bead) than yellow SPLAT with magnesium oxide (18 ± 2) or yellow SPLAT containing a combination of magnesium oxide and barium sulfate (23 ± 2 sheaths/bead) (*F*_3.79_ = 5.25; *P* = 0.002, *n* = 20). Therefore, under low-UV conditions, fewer salivary sheaths were found on yellow SPLAT containing magnesium oxide. The opposite effect was observed for white SPLAT containing magnesium oxide or magnesium oxide + barium sulfate. Within the white SPLAT treatments, significantly more salivary sheaths were present on white SPLAT containing magnesium oxide or magnesium oxide + barium sulfate (*F*_3.79_ = 28.3; *P* < 0.0001, *n* = 20) than on control white SPLAT or white SPLAT with barium sulfate (Fig. 5A). Overall, psyllids probed more often when magnesium oxide alone or magnesium oxide + barium sulfate were present in beads of white SPLAT.

**Table 1:** Effect of SPLAT color and reflectant blend type on mean (±SEM, *n* = 20) no. of salivary sheaths secreted by *Diaphorina citri* adults in SPLAT choice assays.

UV conditions	Effect	Color		Reflectant blend				*F*-value	*P* > *F*
		Yellow	White	Blank	BaSO4	MgO	MgO + BaSO4		
Partial UV	Color	29.2 ± 1.7	9.9 ± 1.7					62.7	<0.001
	Blend type			22.2 ± 2.4	19.3 ± 2.4	17.9 ± 2.4	18.7 ± 2.4	0.60	**0.61**
	Interaction							13.09	<0.0001
UV Chamber	Color	28.0 ± 1.5	11.2 ± 1.5					66.6	<**0.0001**
	Blend type			4.8 ± 2.0	4.7 ± 2.0	36.7 ± 2.1	32.2 ± 2.1	70.2	<**0.0001**
	Interaction							10.7	<**0.0001**
Sunlight	Color	6.8 ± 0.4	2.2 ± 0.4					59.9	<**0.0001**
	Blend type			1.75 ± 0.6	2.3 ± 0.6	7.6 ± 0.6	6.3 ± 0.5	23.8	<**0.0001**
	Interaction							10.1	<**0.0001**

Under higher UV conditions (UV chamber), we observed a more pronounced effect of UV reflectance on psyllid probing behavior. Psyllids were more attracted to yellow than white colored SPLAT (*F*_7, 159_ = 66.6; *P* < 0.0001, *n* = 20). An interaction was observed between the SPLAT color and the reflectant blend type (*F*_7, 159_ = 62.7; *P* < 0.0001, *n* = 20) (Table 1). More salivary sheaths were present on yellow SPLAT containing magnesium oxide alone or magnesium oxide + barium sulfate (*F*_3.79_ = 41.1; *P* < 0.0001, *n* = 20) than on beads of yellow SPLAT or SPLAT containing barium sulfate alone (Fig. 5B). Significantly more salivary sheaths were counted on beads of white SPLAT containing magnesium oxide (*F*_3.79_ = 38.3; *P* < 0.0001, *n* = 20) than on other white SPLAT treatments (Fig. 5B). Greater psyllid probing activity was observed on SPLAT beads containing magnesium oxide or magnesium oxide + barium sulfate, especially with yellow SPLAT, than on blank SPLAT or SPLAT with barium sulfate alone.

Under unfiltered natural sunlight conditions, psyllids were more attracted to yellow than white colored SPLAT (*F*_7, 159_ = 66.6; *P* < 0.0001, *n* = 20). An interaction was observed between SPLAT color and the reflectant blend type (*F*_7, 159_ = 59.9; *P* < 0.0001, *n* = 20) (Table 1). More salivary sheaths were found on yellow SPLAT beads containing magnesium oxide (12 ± 1 sheaths/bead) and magnesium oxide + barium sulfate (9 ± 2 sheaths/bead) than on beads of yellow SPLAT (2 ± 0.4 sheaths/bead) or yellow SPLAT containing barium sulfate (4 ± 1 sheaths/bead) treatments (*F*_3.79_ = 18.15; *P* < 0.0001, *n* = 20) (Fig. 5C). White SPLAT beads containing magnesium oxide + barium sulfate also received significantly more salivary sheaths than other white SPLAT treatments (*F*_3.79_ = 8.73; *P* < 0.0001, *n* = 20) (Fig. 5C). Our results indicated that the compounds tested increased UV reflectance from surfaces of SPLAT under natural sunlight thereby increasing attraction of psyllids towards treated yellow SPLAT beads.

### Choice assay to study the synergistic effect of magnesium oxide with phagostimulant blend

**Figure 6:**
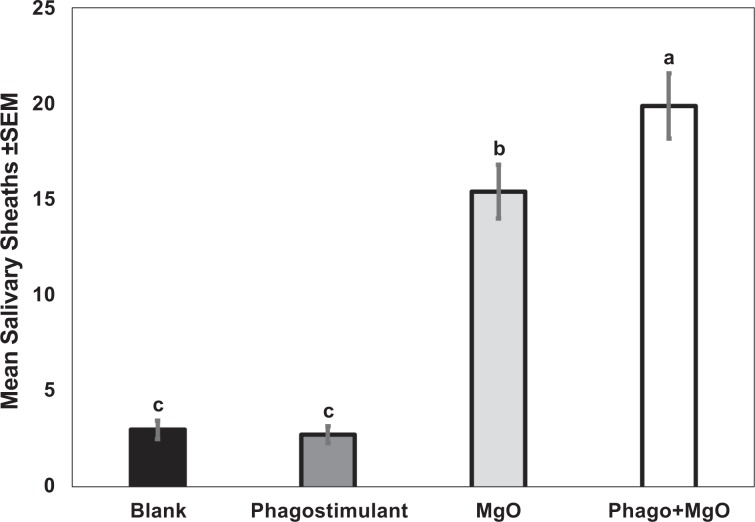
Mean number of salivary sheaths secreted by *D. citri* adults on yellow SPLAT beads containing MgO alone, phagostimulant blend or a combination of MgO + phagostimulant under UV chamber light conditions. Means were compared by Tukey’s HSD following a significant ANOVA. Treatments having no letters in common are significantly different (*α* = 0.05).

Behavioral probing choice assays were performed to investigate a possible synergistic effect of combining magnesium oxide with a chemical phagostimulant blend on behavior of *D. citri*. More salivary sheaths were present on SPLAT beads containing magnesium oxide + phagostimulant blend than on magnesium oxide alone, phagostimulant alone or the yellow blank SPLAT (*F*_3.127_ = 90.54; *P* < 0.0001, *n* = 32) (Fig. 6). Under UV conditions, more psyllids were attracted to yellow SPLAT containing magnesium oxide, and produced significantly more salivary sheaths than with blank yellow SPLAT. However, the presence of phagostimulant along with the magnesium oxide appeared to cause a synergistic effect on psyllid behavior resulting in significantly more salivary sheaths than either of these two treatments presented alone.

## Discussion

It has long been understood that visual cues are predominantly responsible for the orientation of hemipterans such as aphids^34^ and psyllids to their host plants^2,23^. Attraction by the psyllid to wavelengths perceived as yellow or lime-green in the human visual spectrum has been well documented, and is the basis for the use of the “yellow or lime-green” sticky trap for monitoring psylloids^35,36^ including *D. citri*^37^. Prior studies reported the relative importance of olfactory and gustatory cues in the host selection behavior of psyllids in combination with visual cues^2,6,38,39^. Candidate host odor volatiles were less attractive to psyllids, if at all, in the absence of visual cues^2,9^. Setamou *et al*.^10^ reported that *D. citri* did not differentiate between host and non-host plants under dark conditions and showed a stronger response to illuminated vs. darkened host plants. George *et al*.^6^ first reported large and consistent antennal responses by *D. citri* to volatiles such as formic and acetic acids produced as breakdown products when common plant odor compounds such as citral and ocimene come into contact with atmospheric oxygen even though the parental compounds have no antennal activity. Subsequently, Lapointe *et al*.^38^ discovered that formic and acetic acids as well as para-cymene induce increased probing by *D. citri* when incorporated into a wax substrate. All of these studies have revealed the importance of light and visual cues in psyllid orientation towards host plants. Effective and sustainable management strategies that exploit psyllid behavior, such as attract-and-kill, are needed to combine with existing cultural management practices such as reflective mulches.

In field trials, *D. citri* populations were lower on visual targets (young citrus plantings) obscured by color contrasts resulting from reflective mulch^31^. Reflective mulches have been shown to alter insect communities on a variety of crops by disrupting normal landing and take-off cues^40–42^. Reflective mulches may change insects’ visual field by reflecting short-wavelengths (UV) from below foliage thus effectively changing the visual signature of ground from normal long-wavelengths (green-yellow) to abnormal short-wavelengths associated with the sky. However, the visual targets investigated here are characterized by both short (UV) and long (green-yellow) wavelength color. As such, they function as attractive point sources to *D. citri*^43^ and can be used for trapping or attract-&-kill devices.

Magnesium oxide and barium sulfate were previously reported as inert compounds with high UV reflective properties^44–47^. No prior studies have reported how the UV reflective properties of such compounds influence the attraction or probing behaviors of insects. We found here that UV reflectance from the surface of white or yellow SPLAT beads could be dramatically increased by addition of magnesium oxide and barium sulfate powder (Figs. 1B, 2B). Addition of magnesium oxide, an odorless white powder, to the decoy yellow sticky traps increased UV reflectance from the surface of traps (Fig. 3B). Attractiveness of yellow traps increased with increasing amount of magnesium oxide incorporated (Fig. 4). Our previous studies^38^ reported that *D. citri* adults were more attracted to yellow SPLAT compared with white SPLAT, and similar results were observed in probing assays described here. Under low UV conditions, more salivary sheaths were deposited in yellow compared with white SPLAT. In assays conducted in a UV chamber, the presence of magnesium oxide alone or in combination with barium sulfate significantly increased UV reflectance from beads of yellow SPLAT, and caused a significant increase in psyllid attraction and probing on SPLAT. Magnesium oxide alone induced more probing activity on the yellow SPLAT under both natural sunlight and within UV chambers. (Fig. 5B,C). Furthermore, psyllids preferred yellow SPLAT beads containing magnesium oxide and magnesium oxide + phagostimulant blend over the control treatment or yellow SPLAT with phagostimulant blend but without the UV reflectant. Based on the above results, magnesium oxide holds greater potential for improving effectiveness the attract-&-kill technology under development here.

Attract-and-kill devices or traps based on psyllid visual and gustatory behavior could be a valuable addition to practices currently available for management of *D. citri*^48^. Traps combining yellow color (long-range wavelength) with UV (short-range wavelength) reflectance from magnesium oxide should provide an effective combination of visual cues to promote both long-range attraction and sustainted probing behavior by psyllids. To initiate feeding, psyllids produce a gelling saliva that forms a salivary sheath, which surrounds the stylets during subsequent probing and feeding^38^. Previous studies demonstrated that a phagostimulant blend of formic acid, acetic acid and para-cymene increased the number and size of salivary sheaths secreted by adult *D. citri* on beads of yellow SPLAT^38^. Here, we combined the reflectance properties of magnesium oxide with the phagostimulant blend to further enhance psyllid attraction to SPLAT beads compared with attraction to beads containing the phagostimulant alone (Fig. 6). By combining compounds that enhance UV reflectance with a phagostimulant, the frequency and duration of *D. citri* probing can be enhanced to increase exposure to insecticide in or on attract-and-kill devices and subsequent mortality.

Compounds such as magnesium oxide and barium sulfate are highly reflective across the insect and human visual spectra^44^. Magnesium oxide (periclase) is a white, fine powder with high UV reflectance and is used as a reflectance standard^45,46^. It is a divalent oxide that can have surface color centers serving as electron traps following UV exposure; which may explain its UV reflectance^47^. Barium sulfate also has high intrinsic reflectance of UV and visible wavelengths. It is a frequently used pigment in ultraviolet-reflective coatings^49^ and also a reflectance standard because of its high reflectance in both UV and visible spectra^46^. Molecules such as magnesium oxide and barium sulfate offer several advantages over other methods tested for modifying psyllid behavior with visual cues. For example, they are inert and do not require a power source to operate as do LEDs. Here, we show that attract-and-kill devices designed to kill visually oriented insects with piercing/sucking mouthparts can be enhanced by increasing UV reflectance of device surfaces. Our current efforts are focused on exploiting this behavioral response for development of an attract-and-kill device for practical management of this pest.

## Materials and Methods

### Asian citrus psyllids

An uninfected (CLas-free) colony of *D. citri* was maintained at the U.S. Horticultural Research Laboratory, Fort Pierce, FL. The colony was routinely tested to confirm that it was HLB-negative using previously reported PCR methods^50^. Psyllids were reared on seedlings of a susceptible host, *Citrus macrophylla* Wester, and maintained at 28 °C, 14:10 L:D. All insects used for bioassays were adults between 8 and 10 days old; sex ratio was approximately 50:50. All cage experiments were performed in a temperature (26 °C) and humidity (60–65% RH) controlled walk-in chamber under 14:10 L: D conditions. The *D. citri* used for the yellow sticky trap assays were reared in a different greenhouse under similar conditions and protocols at the US Department of Agriculture, Agricultural Research Service, Center for Medical, Agricultural, and Veterinary Entomology in Gainesville, Florida. The host plant used for rearing *D. citri* in Gainesville was orange jasmine, *Murraya paniculata* (Linnaeus). The greenhouse was climate controlled at 29 ± 3 °C under a photoperiod of 16:8 L:D using natural light and metal halide lamps. Plants were watered 3 times weekly and fertilized once a month with one tsp of Milorganite® plus (20-20-20 fertilizer solution). As in Fort Pierce, FL, both rearing plants and randomly selected psyllids from the colony were verified to be free of CLas infection using previously described PCR methods^50^. Adult *D. citri* used for assays from the Gainesville colony represented a mix of ages and physiological states.

### Measurement of UV reflectance from magnesium oxide and barium sulfate

The irradiance spectra of various artificial light sources used in SPLAT probing assays and solar radiation was measured using a concave grating spectrometer (UV-VIS BLACK-Comet, StellarNet Inc, Tampa, FL) over the spectral range of 350–700 nm. The light sources were a standard fluorescent bulb (F40T12/CW Plus, 40 Watt, Philips, USA), metal halide-sodium bulbs (GE Saf-T-Gard Multivapor Quartz Metal Halide ED37, MVT400/I/U; and GE Lucalox Sodium LU400/DX), and unfiltered, natural sunlight. A second concave grating spectrometer was used for the reflectance spectra that had a spectral range of 300–700 nm (UV-VIS BLACK-Comet, StellarNet Inc, Tampa, FL). The reflectance spectra of sticky traps/visual targets used in indoor bioassays and field trials was obtained with the aid of a light source that had a spectral range of ultraviolet and visible light (Deuterium-Tungsten Halogen Light Source, StellarNet Inc, Tampa, FL). A reflectance probe connected to the light source and possessing six illuminating fibers and one read fiber (RPH1 Reflectance Probe, StellarNet Inc, Tampa, FL) was mounted on a holder (RPH1, StellarNet Inc, Tampa, FL) and maintained at a 45° angle at a set distance of 1.9 cm from the sticky traps/visual targets. All measurements were standardized using a white reflectance standard (RS-50, StellarNet Inc Tampa, FL) and a dark reflectance standard obtained by turning off the shutter in the light source. For each measurement of visual targets, two measurements were obtained and then averaged. Only one measurement was performed to quantify the reflectance spectra of the powdered form of magnesium oxide and barium sulfate.

### Visual attraction to yellow sticky cards with different rates of magnesium oxide

All traps consisted of colored sticky cards (10 × 10 cm) prepared from foamboard and painted with selected colors on one side. A hanger for traps consisted of a large paper clip with one end straightened to embed into the foamboard in the center top. The other end was hooked into screens at the back-top seam of the cage using safety pins. Traps were painted with a base white coat (Zinzer 123 primer, Rust-o-leum, Vernon Hills, IL) and then coated with a UV-reflecting yellow bird decoy paint (ReelWings Decoy Co., Inc., Fargo, ND). Sticky adhesive (Tangle-trap®, Tanglefoot, Grand Rapids, MI) was thinly coated on the traps to collect psyllids. Four rates (0 mg, 0.25 mg, 0.5 mg and 1.25 mg) of MgO were compared by mixing with the adhesive before applying on to the sticky cards. The powder was weighed and evenly admixed into the sticky adhesive before application on the traps.

Assays were conducted in the laboratory at 29 ± 2 °C and 40–50% RH under illumination from metal halide lamps (150 W) in digital ballasts. Multi-choice assays were conducted in large screened cages (MegaView Science, Taichung, Taiwan) that were 45 cm tall × 90 cm long × 45 cm wide. Cages had screened sides and clear plastic tops. Traps to be tested were placed equidistantly between cage sides and other traps. The surface of one side of the traps was covered with clear sticky film (Alpha Scents, West Linn, OR). About 100–120 psyllids were released into the cage after traps were placed in the cages. Assays lasted 5 h with psyllids released into cages 0800 hr. After assay completion, traps were removed from the cage and remaining psyllids were aspirated, counted, and sexed. Traps were examined under 10-40x magnification to sex and count psyllids. Data are presented as percentage of total responding psyllids that were caught on each trap. Assays were replicated 12 times. Data were analyzed using analysis of variance (ANOVA) followed by Tukey’s HSD for mean comparisons.

### Effect of magnesium oxide and barium sulfate on probing by *D. citri* under different UV conditions

A completely randomized choice assay was used to study the probing of *D. citri* in response to chemosensory stimuli (odorants and/or tastants) as described previously^6,38,51^. This assay measured insect orientation within a screened cage to combinations of color and texture, and subsequent probing behavior that may result from a combination of olfaction and gustation upon contact with a wax substrate containing magnesium oxide or barium sulfate. Test compounds were incorporated into a slow-release wax matrix (SPLAT™, ISCA Technologies Inc., Riverside, CA) and offered to caged *D. citri* adults in beads of yellow or white SPLAT. A yellow wax substrate was prepared by adding 6 µl of green food coloring (McCormick & Co., Inc., Hunt Valley, MD, USA) to 10 gm of white SPLAT provided by the manufacturer resulting in a yellow-green mixture. Yellow or white stocks were then combined separately with magnesium oxide/barium sulfate powders in a vortex rotor for 5 min. Mixtures were 1% by weight with 100 mg of magnesium oxide or barium sulfate added to 10 gm of SPLAT. One ml of each treatment (white or yellow SPLAT with or without magnesium oxide/barium sulfate) was applied as narrow strips of beads (2.0 × 0.5 × 0.1 cm) to 6 glass cover slips (22 × 22 mm, Fisherbrand Microscope Cover Glass 12-542-B). Each cover slip received approximately 0.17 ml of wax, which was air dried for 18 h. A total of eight treatments were tested as a choice experiment in the initial trials. SPLAT bead treatments compared were: white, blank control; white, magnesium oxide; white, barium sulfate; white, magnesium oxide + barium sulfate; yellow, blank control; yellow, magnesium oxide; yellow, barium sulfate; yellow, magnesium oxide + barium sulfate. The beads were placed in a completely randomized pattern with 5 replications on the floor of a cubical cage (60 × 60 × 60 cm, BioQuip, San Diego, CA). Cages were replicated 4 times and treated as blocks. The same design was used in each cage but with unique randomizations of treatments within each cage.

Cohorts of 250 8- to 10-d-old *D. citri* adults were starved for 3 h and then released into each cage and allowed to move freely and probe into the wax beads within the cage for 22 h. Cages were held in a temperature and humidity controlled incubator at 26 °C, 75% RH and continuous light. Experiments were performed under various low and high UV (UV chamber), as well as, unfiltered, natural sunlight conditions to quantify the effect of UV reflectance from the surface of SPLAT beads on psyllid attraction and probing behavior. Experiments under low UV conditions were performed under fluorescent lights (F40T12/CW Plus, 40 Watt, Philips, USA). SPLAT probing experiments under complete UV conditions were performed in a UV chamber containing sodium vapor lamps and metal halide lamps (GE Saf-T-Gard Multivapor Quartz Metal Halide ED37, MVT400/I/U; and GE Lucalox Sodium LU400/DX). The same experiments were performed outdoors under unfiltered, natural sunlight conditions during tdaylight hours (11:00-17:00). SPLAT beads from the 8 treatments were placed in a completely randomized pattern with 5 replications on the floor of a cubical cage (60 × 60 × 60 cm, BioQuip, San Diego, CA). Cages were replicated 4 times and treated as blocks. Cohorts of 250 8- to 10-d-old *D. citri* adults were starved for 3 h and then released into each cage and allowed to move freely and probe into the wax beads for 6 h. The time was limited to 6 hours of highest UV daylight.

To visualize salivary sheaths produced by feeding attempts on the wax beads, cover slips were removed from the cages and beads were stained with Coomassie blue dye for 60 sec for yellow SPLAT beads and 45 sec for white beads, washed in water and allowed to air-dry^6,38,51^. The number of salivary sheaths in each bead was counted under a stereomicroscope at 50 X magnification. The difference in number of salivary sheaths between treatments were analyzed by ANOVA followed by Tukey’s HSD for comparison of means.

### Effect of phagostimulant blend with magnesium oxide on probing choice

Phagostimulant compounds can induce feeding and such compounds could act as nutrients or token stimuli for insects. Our earlier studies have reported that a phagostimulant blend of *formic acid: acetic acid: p-cymene* in the ratio *3.5:1.6:1* can increase probing behavior by *D. citri* as measured by greater deposition of salivary sheaths^38^. In this experiment, we tested how addition of magnesium oxide to SPLAT containing phagostimulant blend affects probing behavior and salivary sheath secretion by psyllids. A choice assay was performed using yellow SPLAT beads containing magnesium oxide with and without phagostimulant blend. Experiments were performed in a temperature and humidity controlled UV chamber at 26 °C, 75% RH and continuous light. Treatments compared were: yellow SPLAT beads, yellow beads containing magnesium oxide, yellow beads containing the phagostimulant blend, and yellow beads containing magnesium oxide + the phagostimulant blend. One ml of each treatment (yellow SPLAT with or without MgO/phagostimulant blend) was applied as a narrow strip of beads (2.0 × 0.5 × 0.1 cm) to 6 glass cover slips (22 × 22 mm, Fisherbrand Microscope Cover Glass 12–542-B). Each cover slip received approximately 0.17 ml of wax and 0.67 μl of the phagostimulant blend, or 1.6 mg of magnesium oxide or a combination of both depending on the treatments. The beads were air-dried for 2 h prior to assays. Treatments were arranged in a completely randomized pattern on the floor of a cubical cage (60 × 60 × 60 cm, BioQuip, San Diego, CA). Cohorts of 250 8- to 10-d-old *D. citri* adults were starved for 3 h and then released into each cage and allowed to move freely and probe into the wax beads within the cage for 22 h. Cages were replicated 4 times and treated as blocks. Means were compared by Tukey’s HSD following a significant ANOVA.

## Supplementary information

Supplementary information.

Supplementary information 2.

Supplementary information 3.

Supplementary information 4.

## Data Availability

The datasets generated during and/or analyzed during the current study are available from the corresponding author upon request.
