# Supplementary material for: UV reflective properties of magnesium oxide increase attraction and probing behavior of Asian citrus psyllids (Hemiptera: Liviidae)
Source: Sci Rep. 2020 Feb 5;10:1890. doi: 10.1038/s41598-020-58593-4 (PMC7002715; doi:10.1038/s41598-020-58593-4)
Supplement: Supplementary file 1 — Supplementary information. [file 41598_2020_58593_MOESM1_ESM.docx]

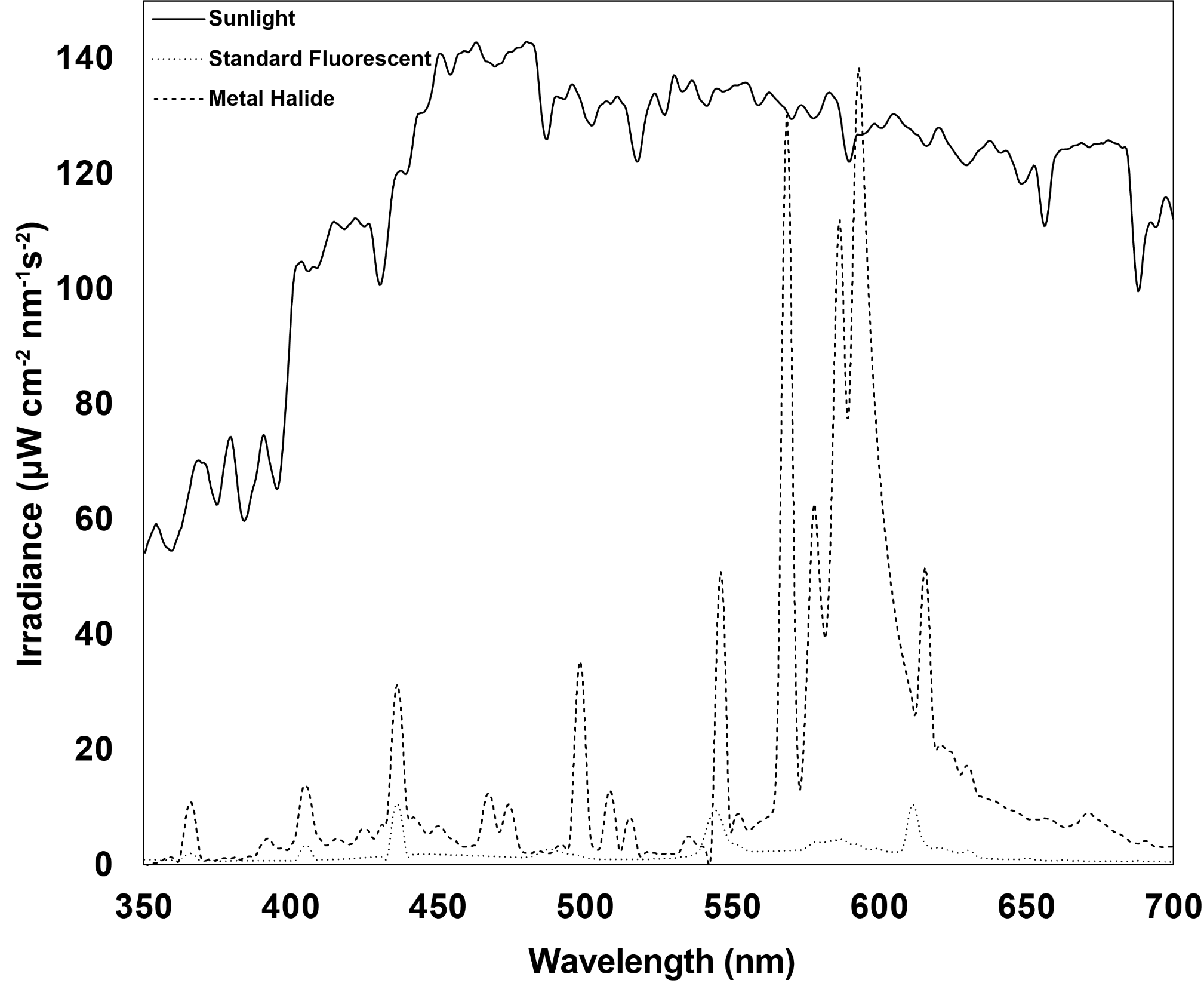


**Figure S1.** Irradiance spectra of the three different light sources under which sticky trap assays were conducted. The three light sources depicted are as follows 1) Sunlight (dash and dot line). 2) Fluorescent (dotted line) 3) Metal Halide Lamp (dashed line).
