# Supplementary material for: UV reflective properties of magnesium oxide increase attraction and probing behavior of Asian citrus psyllids (Hemiptera: Liviidae)
Source: Sci Rep. 2020 Feb 5;10:1890. doi: 10.1038/s41598-020-58593-4 (PMC7002715; doi:10.1038/s41598-020-58593-4)
Supplement: Supplementary file 2 — Supplementary information 2. [file 41598_2020_58593_MOESM2_ESM.docx]

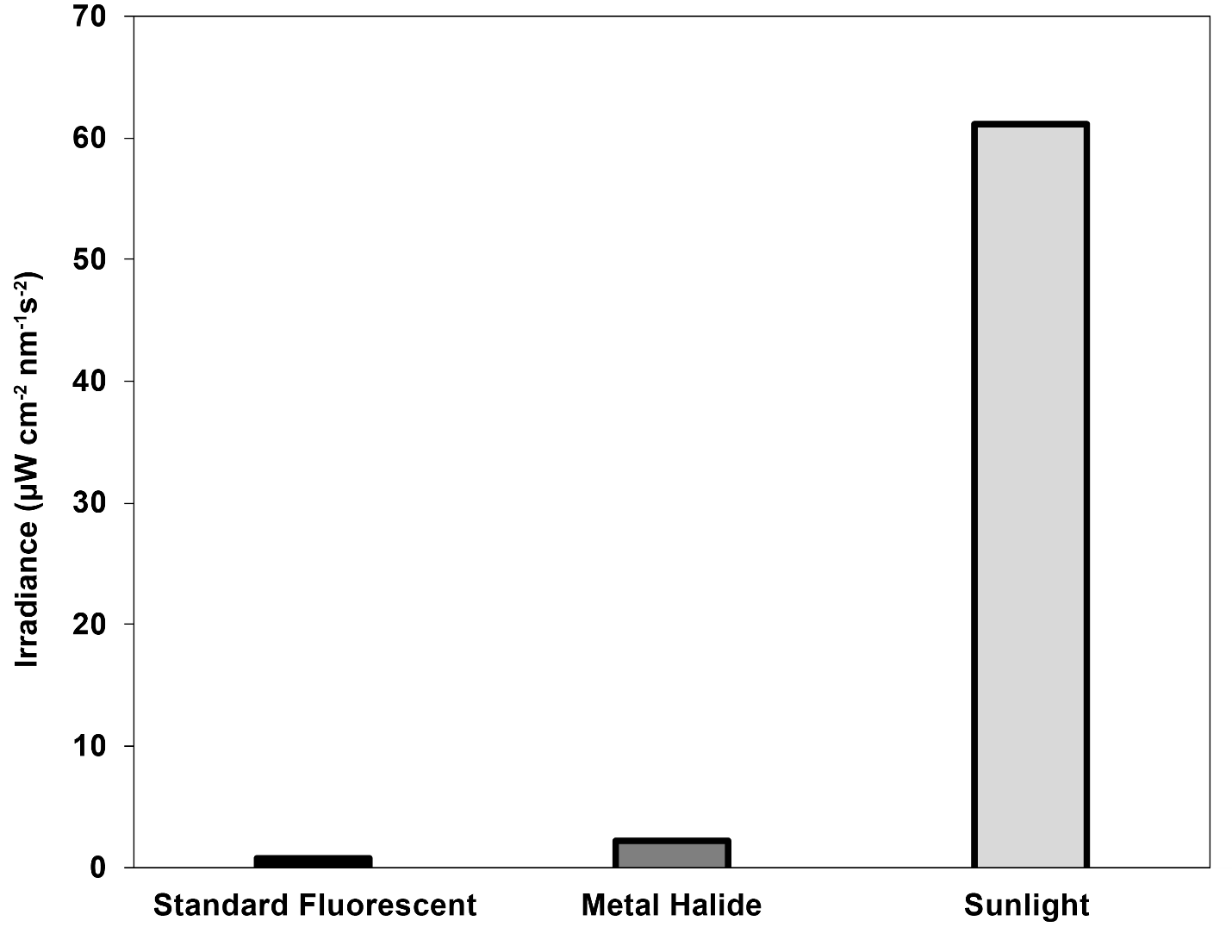


**Figure S2**. Irradiance spectra for each of the light sources in the bioassays with respect to their amount of ultraviolet radiation. The mean ultraviolet irradiance spectra are depicted between a range of 350 to <400 nm.
