# Supplementary material for: UV reflective properties of magnesium oxide increase attraction and probing behavior of Asian citrus psyllids (Hemiptera: Liviidae)
Source: Sci Rep. 2020 Feb 5;10:1890. doi: 10.1038/s41598-020-58593-4 (PMC7002715; doi:10.1038/s41598-020-58593-4)
Supplement: Supplementary file 3 — Supplementary information 3. [file 41598_2020_58593_MOESM3_ESM.docx]

**Figure S3.** Reflectance spectra of MgO (solid line) and BaSO_4_ powder (dotted line).
