# Supplementary material for: UV reflective properties of magnesium oxide increase attraction and probing behavior of Asian citrus psyllids (Hemiptera: Liviidae)
Source: Sci Rep. 2020 Feb 5;10:1890. doi: 10.1038/s41598-020-58593-4 (PMC7002715; doi:10.1038/s41598-020-58593-4)
Supplement: Supplementary file 4 — Supplementary information 4. [file 41598_2020_58593_MOESM4_ESM.docx]

**Supplementary figures**

**Figure S1.** Irradiance spectra of the three different light sources under which sticky trap assays were conducted. The three light sources depicted are as follows 1) Sunlight (dash and dot line). 2) Fluorescent (dotted line) 3) Metal Halide Lamp (dashed line).

**Figure S2**. Irradiance spectra for each of the light sources in the bioassays with respect to their amount of ultraviolet radiation. The mean ultraviolet irradiance spectra are depicted between a range of 350 to <400 nm.

**Figure S3.** Reflectance spectra of MgO (solid line) and BaSO_4_ powder (dotted line).
